# The Nature of Non-Arrhenius Kinetics in the Heat Denaturation of Proteins

**DOI:** 10.3390/ijms27146449

**Published:** 2026-07-20

**Authors:** Alexey V. Baklanov, Alexey O. Yanshin

**Affiliations:** 1 Voevodsky Institute of Chemical Kinetics and Combustion SB RAS, 3 Institutskaya Street, 630090 Novosibirsk, Russia; yanshin@g.nsu.ru; 2 Department of Physics, Novosibirsk State University, 1 Pirogova Street, 630090 Novosibirsk, Russia

**Keywords:** proteins, unfolding, free-energy profile, dry molten globule, heat denaturation, non-Arrhenius kinetics

## Abstract

The nature of non-Arrhenius kinetics of protein unfolding is investigated in this study. Free-energy profiles along the reaction coordinate of protein unfolding are built in a wide temperature interval. These profiles reveal the temperature-dependent contribution of the intermediate assigned to be the dry molten globule (*DMG*) state, stabilized by the entropy gain provided by the loose framework of extended hydrogen bonds. The revealed *DMG* state with a loose pseudo-secondary structure of protein provides a funnel-shaped free-energy landscape, which is a central point of the folding mechanism, rationalizing Levinthal’s paradox. The rate constants of the elementary steps of the unfolding process are calculated according to Transition State Theory. The strong temperature dependence of the Arrhenius parameters for the rate constants of the elementary steps of the unfolding process, and the negative activation energy of the folding process are explained. The main factor influencing the non-Arrhenius behavior of the rate constants is the strong temperature-dependent shift in the location of the *DMG* and Transition State along the reaction coordinate. The Arrhenius plot for the calculated rate constant for heat denaturation of the protein in a wide temperature range (270–600 K) is built. Its “convex” shape and the sharp drop in the values of the Arrhenius parameters at high temperatures are in very good agreement with the experimentally observed dependencies.

## 1. Introduction

Protein denaturation results in the loss of the native structure and protein functionality. Temperature is a frequent factor involved in the loss of functionality. Heating leads to thermal inactivation of microorganisms (bacteria, viruses, etc.), which is used for food and biomaterial processing through thermal therapy. Temperature lowering is used in cryosurgery and cryotherapy. All these applications have led to academic interest in the temperature dependence of protein denaturation kinetics. The temperature dependence of the rate constant is usually empirically described with the Arrhenius equation:

(1) $$k=A\times e^{-\frac{E_{a}}{RT}}$$

or, more specifically, with the Transition State Theory (TST) equation [1]:

(2) $$k=\frac{k_{B}T}{h}\cdot e^{-\frac{∆F^{\neq }}{RT}}=\frac{k_{B}T}{h}\cdot e^{\frac{∆S^{\neq }}{R}}\cdot e^{-\frac{∆H^{\neq }}{RT}},$$

where $∆F^{\neq }=∆H^{\neq }-T\cdot ∆S^{\neq }$ is the free energy of the Transition State, corresponding to the maximum of the free energy on the reaction coordinate; $∆S^{\neq }$ and $∆H^{\neq }$ are the entropy and the enthalpy of activation. Large amounts of data are reported in the literature on the parameters (*A* with $E_{a}$ or $∆S^{\neq }$ with $∆H^{\neq }$) of Equations (1) and (2) for heat denaturation [2,3,4,5,6,7,8]. Qin et al. [6] summarized the experimental literature data presented in the review by He and Bischof [5] and reported that the activation energy $E_{a}$ in various protein systems varies between 100 and 800 kJ/mol (about 25–200 kcal/mol), and the preexponential factor *A* between 10^9^ and 10^129^ s^−1^. Even higher numbers for *A* and $E_{a}$ (up to 10^218^ s^−1^ and about 310 kcal/mol, respectively) are reported in the data reviewed by Wright [7]. Denaturation is supposed to proceed via unfolding of the secondary or higher-order structure of protein. The above-presented experimentally measured values of the *A-*factor up to 10^129^ s^−1^ and even up to 10^218^ s^−1^ are much higher than the numbers observed for the unimolecular reactions proceeding via the rearrangement of covalent bonds, which are usually close to the typical value of the molecular vibrational frequency ν = 10^13^–10^14^ s^−1^ (see textbook [9]). Sarkar et al. considered values of the *A*-factor higher than 1 × 10^14^ s^−1^ to be “unphysical”, keeping in mind that the *A* value is the fastest possible unfolding rate (at an infinite temperature) [10]. Besides the exotic values of Arrhenius parameters, the data for protein unfolding also demonstrate non-Arrhenius behavior, which usually corresponds to a decrease in the Arrhenius parameters with increasing temperature, resulting in convex Arrhenius plots [6,10,11,12,13,14]. The presence of a “convex” shape in the Arrhenius plots in nonbiological and biochemical kinetics was previously generally attributed to complex (multistep) mechanisms [11,12]. It makes sense to pay special attention to the results of paper [10], where the nature of the contributing denaturation mechanisms is addressed. The authors of paper [10] presented the results for the denaturation rates of several proteins obtained over a very wide temperature range (300–600 K). The expansion of the temperature range above the boiling point of water to about 600 K was achieved in paper [10] using an earlier developed technique of nanosecond plasmonic heating [15]. The observed non-Arrhenius temperature dependence was interpreted as resulting from the contribution of two (low- and high-temperature) mechanisms [10]. The low-temperature mechanism of this protein deactivation was concluded to be limited by the reaction with activation energy near 40 kcal/mol and a preexponential factor of about 10^24^–10^25^ s^−1^ [10]. In turn, the high-temperature mechanism with an activation energy of 2–3 kcal/mol and a preexponential factor near 10^9^ s^−1^ was concluded to be limited by diffusion [10]. In our current work, we present an approach which allows us to calculate the temperature dependence of the rate constants of protein denaturation in a wide temperature range and compare it with the experimental data.

In our recent paper, the nature of very high values of the preexponential factor for protein unfolding was explained within Transition State Theory with consideration of unfolding as the breaking of multiple interchain hydrogen bonds [16]. It was shown that the preexponential factor *A* depends on the number of dissociating interchain hydrogen bonds (*H*-bonds) and can have very high values similar to the numbers measured experimentally. The key reason for very high values of *A*−factor is the very loose structure of the frame of extended interchain *H*-bonds in the Transition State of protein unfolding, which provides very high values of the entropy of activation $∆S^{\neq }$ [16]. This model was also applied for calculations of kinetic isotope effects in the unfolding process [17]. In the current work, we apply a more general approach, in which TST calculations for protein unfolding are based on the built free-energy profile along the reaction coordinate. The reaction coordinate corresponds again to simultaneous breaking of the manifold of interchain *H*-bonds. As noted in paper [16], the frame of interchain *H*-bonds consists of a sequence of similar links, containing two nearby *H*-bonds. In order to build the free-energy profile, the profiles of entropy and enthalpy variation along the reaction coordinate for dissociation of hydrogen bonds N−H⋯O=C were obtained with calculations for the formamide dimer dissociation as a model system. Formamide and the formamide dimer are the smallest systems containing interchain *H*-bonds formed by the atoms of peptide (−CO−NH−) groups. Hydrogen bonds N−H⋯O=C in these molecules are considered good models for hydrogen-bond interaction in proteins and peptides [18,19]. The cyclical structure of the formamide dimer, provided by a pair of nearby *H*-bonds, is similar to the structural features of proteins [20]. We have determined the functional form of enthalpy and entropy dependence on the distance of the dissociating *H*-bond. Then, this dependence and literature data for enthalpy and entropy of protein unfolding were used for building the free-energy profile along the reaction coordinate of unfolding. These free-energy profiles allowed us to reveal the presence of the intermediate state and to determine the location and height of the free-energy barriers and calculate the rate constants for the elementary steps of unfolding. The results of these calculations reproduce many experimental observations and allow us to establish the nature of non-Arrhenius kinetics in the heat denaturation of proteins.

## 2. Results

### 2.1. Shape of the Free-Energy Profiles for the Reaction Coordinate of Protein Unfolding

**Figure 1:**
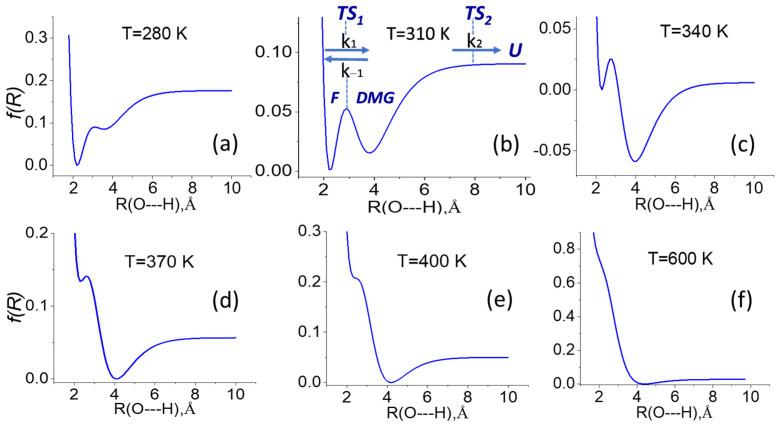
The calculated shape-function f(R) (5) for the free-energy profiles along unfolding reaction coordinate at different temperature (*T*) values. Figure (**b**) contains the scheme of the processes taking place in the unfolding of protein. Region *F* corresponds to the folded structure, *DMG* to the “dry molten globule” structure, and *U* to the unfolded structure of protein (see text). The rate constants and corresponding Transition States *TS*_1_ and *TS*_2_ for interstate transitions are indicated. Zero on *y*-axis in Figures (**a**–**c**) corresponds to the free-energy minimum for the *F* state and (**d**–**f**) for the *DMG* state.

**Figure A1:**
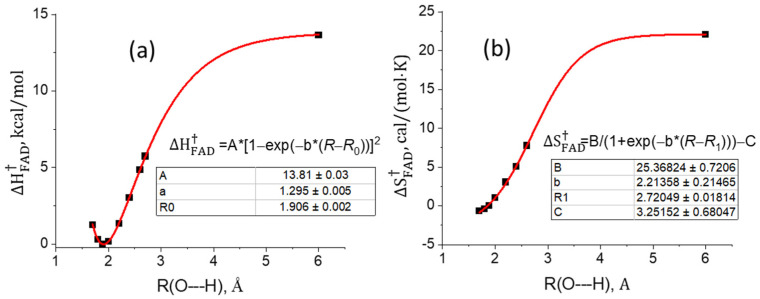
Calculated profiles of the change in (**a**) enthalpy $∆H_{FAD}^{†}(R)$ and (**b**) entropy $∆S_{FAD}^{†}(R)$ of the formamide dimer along the reaction coordinate of the dimer dissociation into monomers at T = 300 K. Points at a long distance of 6 Å are calculated within the Completely Loose Transition State limit (CLTS).

The details of the approach used for quantum-chemical calculations of the structure, energy and vibrational frequencies of the formamide dimer in the course of the *H*-bonds’ dissociation are given in the Methods section. The equilibrium plane structure of this dimer (see Figure 1 of paper [20]) contains two equivalent hydrogen bonds: N−H⋯O=C. Vibration, corresponding to simultaneous symmetric stretching of O⋯H bonds, was considered to be a reaction coordinate R≡R(O⋯H) for the dimer dissociation. Calculated structure, energy and vibrational frequencies of the formamide dimer (FAD) changing along the reaction coordinate were then used for the calculations of the profiles for enthalpy $∆H_{FAD}^{†}(R)$ and entropy $∆S_{FAD}^{†}(R)$ along the reaction coordinate of FAD dissociation. The results are described in Appendix A.1. The obtained results show that the *R*-dependencies $∆H_{FAD}^{†}(R)$ and $∆S_{FAD}^{†}(R)$ for dissociation of the dimer of formamide can be interpolated well with Morse and sigmoidal functions, respectively (see Figure A1 in Appendix A.1). The total enthalpy $∆H^{†}\left(R\right)$ and entropy $∆S^{†}\left(R\right)$ of the manifold of breaking *H*-bonds in protein are the sum of these contributions. Therefore, the *R* dependencies of total enthalpy and entropy of the manifold of breaking *H*-bonds are functionally the same as those for FAD. This allows us to build the interpolation formula for the free-energy profile $∆F^{†}\left(R\right)$ along the reaction coordinate of protein unfolding (see Equation (A6) in Appendix A.2):

$$∆F^{†}\left(R\right)=∆H^{†}\left(R\right)-T\cdot ∆S^{†}\left(R\right)\approx$$

(3) $$\approx ∆H_{\infty }\cdot {\left[1-\exp\left(-1.3\cdot \left(R-2.1\right)\right)\right]}^{2}-T\cdot ∆S_{\infty }\cdot \left[\frac{1.17}{1+exp\left(-2.2\left(R-2.9\right)\right)}-0.17\right],$$

where $∆H_{\infty }$ and $∆S_{\infty }$ are the values of enthalpy and entropy of protein unfolding (correspond to R→∞). Literature data for a linear correlation between enthalpy and entropy of unfolding (see Appendix A.2) allow us to rewrite Equation (3) as follows:

(4) $$∆F^{†}\left(R\right)\approx ∆H_{\infty }\cdot f(R),$$

(5) $${f\left(R\right)=\left[1-\exp\left(-1.3\cdot \left(R-2.1\right)\right)\right]}^{2}-0.9\cdot \frac{T}{300}\cdot \left[\frac{1.17}{1+exp\left(-2.2\left(R-2.9\right)\right)}-0.17\right].$$

Here, the dimensionless function f(R) shows the shape of the free-energy $∆F^{†}\left(R\right)$ profile, which is the same for any value of $∆H_{\infty }$ (any size of the manifold of breaking *H*-bonds). For further calculations, we neglected the temperature dependence of $∆H_{\infty }$ and $∆S_{\infty }$ in the temperature interval considered. We carried out the calculations of the shape-function f(R) for the temperature interval 270–370 K of biological relevance and at higher temperature values achieved in experiments by Sarkar et al. [10]. The profiles f(R) for six temperature values are presented in Figure 1.

These profiles indicate the presence of three states with varied T-dependent contributions. In Figure 1b, their assignment is given. The folded state of protein with equilibrium interchain *H*-bonds is designated in Figure 1b as the *F* state. This state is usually considered to be the native one. The unfolded state (*U*) is attributed to the structure with broken interchain *H*-bonds ($R\left(O\cdots H\right)>R({TS}_{2})$ in Figure 1b). An intermediate structure with extended interchain *H*-bonds is attributed to the so-called dry molten globule (*DMG*), which identifies the expanded form of protein, which does not contain solvent molecules. The presence of this kind of expanded structure on the way from a folded to an unfolded state of protein was first proposed by Shakhnovich and Finkelstein [21]. This structure was supposed to correspond to the short-lived configuration at a maximum of the free-energy barrier. Numerous results, obtained later and indicating the formation of observable expanded intermediate, defined as *DMG*, were discussed in the literature [22,23,24,25,26,27,28,29,30,31,32,33,34,35]. These structures convert to each other via the elementary processes presented in Figure 1b according to the following scheme:

(6) $$F\begin{array}{c} \overset{{\;k}_{1\;}}{\to }\\ \underset{{\;k}_{-1}}{\leftarrow } \end{array}DMG\overset{{\;k}_{2}\;}{\to }U.$$

For further use of Equation (4) in the numerical calculations of the free-energy profiles and the rate constants of the elementary steps of scheme (6), we choose the value of unfolding enthalpy $∆H_{\infty }$ = 50 kcal/mol. This value corresponds approximately to the middle of the range of the experimentally measured unfolding enthalpy dataset for proteins collected by Liu et al. [36]. The influence of $∆H_{\infty }$ variation will be detailed when discussing the results of the calculations.

### 2.2. Constant of Equilibrium F⟺DMG

**Figure 2:**
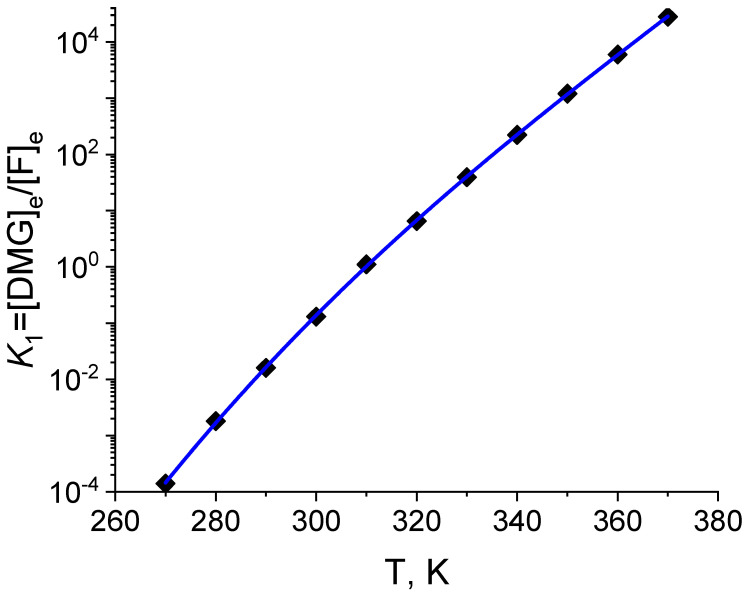
Calculated temperature dependence of the equilibrium constant *K*_1_ equal to the ratio of the equilibrium concentrations of dry molten globule ([*DMG*]_e_) and folded protein ([*F*]_e_). Calculations are carried out with the use of the unfolding enthalpy value $∆H_{\infty }$ = 50 kcal/mol (see text). The result of data fitting by cubic polynomial is shown by the blue line.

The profiles of the shape-function f(R) in Figure 1 show that the relative contributions of the *F* and *DMG* states vary sharply with temperature. The ratio of [*DMG*]_e_ and [*F*]_e_ concentrations in equilibrium, equal to equilibrium constant *K*_1_, was calculated as a function of temperature via Equation (A7), described in Appendix B.1. The results of the calculations presented in Figure 2 demonstrate a very fast rise in *DMG* population with the increase in temperature.

The presented results are obtained with the use of unfolding enthalpy value $∆H_{\infty }$ = 50 kcal/mol. The variation in the $∆H_{\infty }$ value does not change the qualitative shape of presented dependence. The increase in the $∆H_{\infty }$ value makes the slope of this dependence steeper with only a slight shift in the temperature value corresponding to the equilibrium constant value *K*_1_ = 1.

### 2.3. Rate Constants of the Elementary Steps of Protein Unfolding

**Figure 3:**
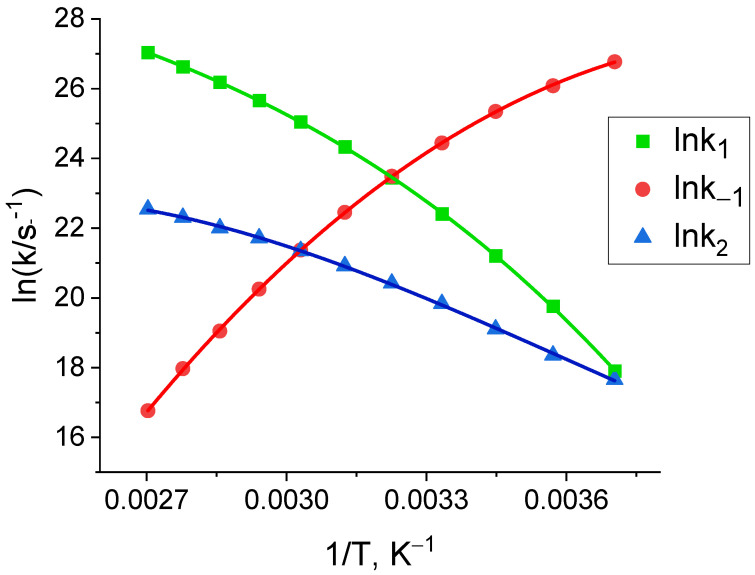
The Arrhenius plots of the calculated rate constants of the elementary steps of the protein unfolding process with denaturation enthalpy of $∆H_{\infty }$ = 50 kcal/mol (see text). The curves are the results of data fitting by cubic polynomials.

The rate constants for the steps of mechanism (6) were calculated with formula (B2), derived from TST Equation (2), as described in Appendix B.2. Temperature dependencies of the calculated rate constants are presented in the Arrhenius coordinates in Figure 3.

**Figure 4:**
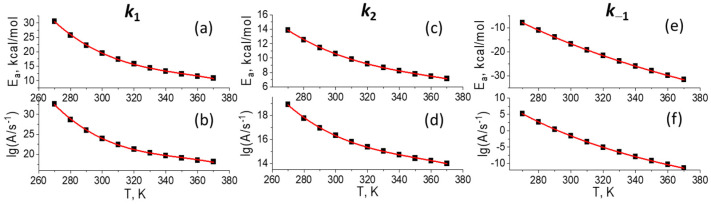
The temperature dependence of the Arrhenius parameters (*E_a_* and lg*A*) for the rate constants *k*_1_, *k*_2_ and *k*_−1_: figures (**a**,**c**,**e**) show activation energy (*E_a_*); figures (**b**,**d**,**f**) show preexponential factor (lg*A*). Red lines are the results of data fitting by cubic polynomials.

All three dependencies presented in Figure 3 are non-linear in the Arrhenius coordinates, which indicates non-Arrhenius behavior of these rate constants. The temperature dependence in the Arrhenius coordinates for all these rate constants are of “convex”-shape. Our results show that even the elementary steps of protein unfolding can demonstrate “convex” temperature dependence. The attempt to describe these dependencies from Figure 3 with the Arrhenius Equation (1) results in a strong dependence of the apparent Arrhenius parameters on temperature, as shown in Figure 4.

**Figure 5:**
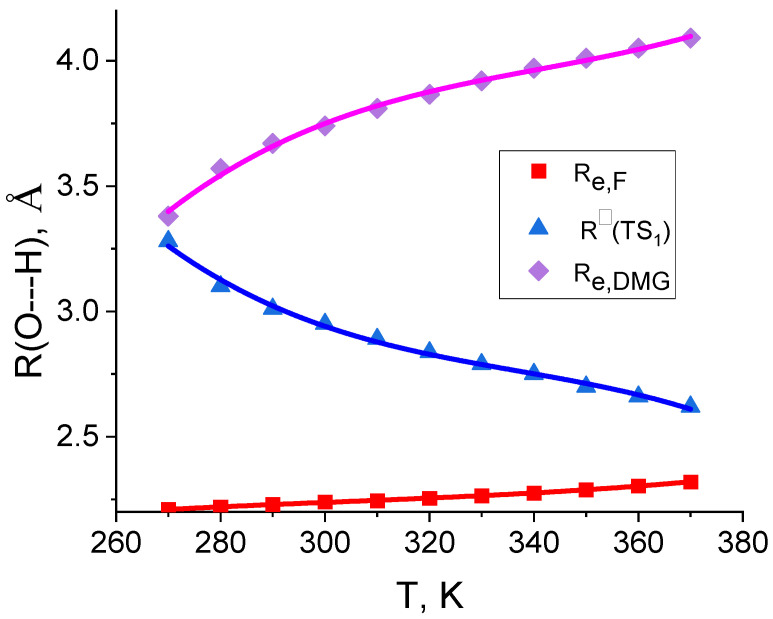
The temperature dependence of *R(O---H)* distances, corresponding to the free-energy minima for folded ($R_{e,F}$) and *DMG* ($R_{e,DMG}$) states, as well as the free energy for Transition State ${TS}_{1}$($R_{TS1}$), shown in Figure 1b. The lines are the results of data fitting by cubic polynomials.

Figure 4b shows very high values of the preexponential factor (lg(*A*/s^−1^) = 32.6) for the rate constant *k*_1_ at low temperature. At higher temperature, up to 370 K, the strong drop in *A*-values takes place. Very exotic temperature dependence is found for the rate constant of the reverse conversion of dry molten globule to folded state *DMG*$\overset{k_{-1}}{\to }$*F*. Besides the strong change in the Arrhenius parameters (see Figure 4e,f), we should highlight the negative activation energy and the preexponential factor values 10^5^−10^−10^ s^−1^, which are “exotically” low as compared with the data for unimolecular reactions proceeding via rearrangement of covalent bonds. These values of the Arrhenius parameters are provided by the large contribution of the entropic term ($-T\cdot ∆S^{\neq }$). Due to comparable contributions of the enthalpic and entropic terms into the free-energy value, the change in temperature provides a very strong effect on the free-energy profile along the reaction coordinate, as shown in Figure 1. An important result of this effect is presented in Figure 5, where the temperature-related changes in the location of the Transition State *TS*_1_ and free-energy minima for *F* and *DMG* states are shown. These changes in location with temperature are followed by variation in enthalpy and entropy of the *TS*, and the *F* and *DMG* states, resulting in the change in the Arrhenius parameters.

The drop in $R^{\neq }(TS_{1})$ from 3.3 to 2.6 Å (see Figure 5) in the considered *T*-interval results in a decrease in activation enthalpy $∆H^{\neq },$ which gives rise to a decrease in $E_{a}$ for *k*_1_ (see Figure 4a). This drop makes the *TS*_1_ structure much more rigid, which leads to a strong decrease in the activation entropy and preexponential factor (lg*A* in Figure 4b) for the process $F\overset{k_{1}}{\to }DMG$. The intervals of *E_a_* and lg*A* variation (see Figure 4c,d) for $DMG\overset{k_{2}\;}{\to }U$ conversion are smaller than those for *k*_1_ because the difference in enthalpy and entropy between *DMG* and *TS*_2_ states is smaller. Exotic *T*-dependence for the rate constant $k_{-1}$ of the process *DMG* → *F* is governed mainly by the shift in *TS*_1_ location. The Transition State *TS*_1_ is located at a smaller *R*-value than the minimum of the *DMG* state (see Figure 1b). Therefore, the enthalpy of *TS*_1_ state is lower than the enthalpy of the *DMG* state. This results in the negative $E_{a}$ values shown in Figure 4e. Therefore, the presence of the free-energy barrier for *DMG* → *F* conversion (see Figure 1) is governed exclusively by the entropy term $-T\cdot ∆S_{-1}^{\neq }$. Here, the $∆S_{-1}^{\neq }$ value is negative in sign because *TS*_1_ is more rigid than the *DMG* state. Negative $∆S_{-1}^{\neq }$ values provide the numbers of *A* = $10^{5}-10^{-10}$ s^−1^, which are unprecedentedly low compared with the “normal” *A* values for unimolecular reactions proceeding via transformation of covalent bonds.

### 2.4. Kinetics of Heat Denaturation in a Wide Temperature Range

**Figure 6:**
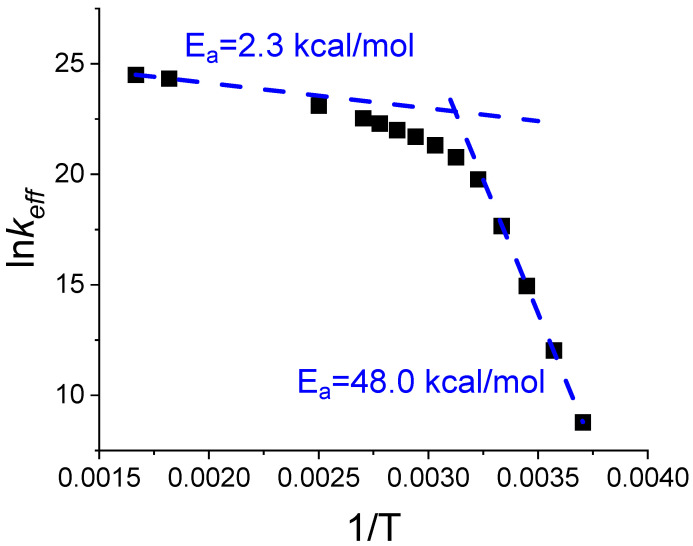
The Arrhenius plot for the calculated rate constant of protein unfolding $k_{eff}=\frac{K_{1}}{1+K_{1}}\cdot k_{2}$ (see text). Calculations are carried out with the use of the unfolding enthalpy value $∆H_{\infty }$ = 50 kcal/mol (see text). Dashed straight lines correspond to the Arrhenius parameters for low- and high-temperature limits.

The calculated rate constants of the elementary steps of mechanism (6) allow us to calculate the rate of heat denaturation, which is usually measured as a rate of the loss of the folded structure $\frac{d[F]}{dt}$. The loss of the *F* state in the heat denaturation process (6) proceeds with F↔DMG equilibration as a first step, followed by conversion DMG→U as a second step. Very fast relaxation to equilibrium F↔DMG proceeds with characteristic time $\tau =\frac{1}{k_{1}+k_{-1}}$, which is always shorter than the time ($\frac{1}{k_{2}}$) of the following conversion DMG→U (see rate constants in Figure 3). The presence of permanent equilibrium between the *F* and *DMG* states is in agreement with literature data [25,26,30,32]. The rate of the *F*-state loss is $\frac{d[F]}{dt}=k_{eff}\cdot [F]$, where $k_{eff}=\frac{K_{1}}{1+K_{1}}\cdot k_{2}$. The temperature dependence of the calculated rate constant of unfolding $k_{eff}$ is shown in Figure 6.

Figure 6 demonstrates a dramatic drop in activation energy from about 48 kcal in the low-temperature limit to about 2 kcal/mol at 600 K. The preexponential factor also drops from $A\approx 10^{43}s^{-1}$ at low temperature to about $10^{11.5}\;s^{-1}$ at high temperature. This sharp drop in the values of Arrhenius parameters is very similar to the experimental data for several proteins summarized by Sarkar et al. in paper [10].

Here, we can repeat that the change in the parameter $∆H_{\infty }$, proportional to the size of the manifold of breaking *H*-bonds, does not change the shape of the free-energy profiles (see Equation (4)). Therefore, the variation in $∆H_{\infty }$ changes the quantitative results but does not change the qualitative picture. For example, the three-time increase in $∆H_{\infty }$ to the value of 150 kcal/mol provides a calculated Arrhenius plot for $k_{eff}$ similar to that in Figure 6. Both the low- and high-temperature limits give activation energy Ea values, which are proportionally (three times) larger than the parameters extracted from Figure 6.

## 3. Discussion

The results obtained show that the applied model of protein unfolding as the simultaneous breaking of multiple interchain *H*-bonds allows us to understand the nature of several important phenomena specific for the process of protein heat denaturation. One of these phenomena is the temperature-dependent contribution of the intermediate, identified earlier as the dry-molten globule state of protein, which plays an important role in both folding and unfolding processes. Other phenomena are non-Arrhenius kinetics of heat denaturation and “exotic” Arrhenius parameters. Below, all these phenomena are discussed in more detail.

### 3.1. Nature of Dry-Molten Globule

The current understanding of the properties of dry-molten globule is based on the summary formulated by Baldwin et al. [27]. According to the picture from paper [27], the *DMG* state was supposed to be stabilized by entropy gain provided by the unlocking of side chains. Structural changes giving rise to the *DMG* state were supposed to proceed at the level of the tertiary structure with conservation of the secondary one [27]. Our results also show that the DMG state is stabilized by entropy gain in the course of unfolding. But the source of this entropy gain is the loosening of the framework of *H*-bonds provided by their extension in the course of unfolding. Our results also show that the *DMG* structure should appear due to expansion of the secondary structure as well. The entropy gain is governed not by a level of structure (secondary or higher) but by the properties of the *H*-bonds. Important properties are the comparable weights of enthalpy ($∆H^{†}\left(R\right))$ and entropy ($T\cdot ∆S^{†}\left(R\right))$, characteristic contributions of the dissociation of *H*-bonds in a temperature interval of biological relevance [16,37]. Therefore, we can expect that the local minima on a free-energy profile can take place for the structures provided by expansion of the structure of any level (secondary, tertiary, quaternary) when this structure is provided by *H*-bonds.

We should also pay special attention to the DMG state corresponding to a highly loosened secondary structure of protein. We think that this state plays a very important role in the folding process. Here, we should first refer to the current picture of the protein folding mechanism, as described in the text book by Bahar, Jernigan and Dill [38]. This mechanism is formulated as an answer to Levinthal’s paradox [39], which puzzles over fast search of the native state in the protein folding process. The mechanism described involves the postulated but not specified intermediate state with a partially assembled structure of protein. This intermediate state corresponds to a broad funnel on a free-energy landscape. The slope of this funnel provides the directed shift in the folding process to the native structure [38]. It is worth noting here that the DMG state under discussion has the properties of the intermediate state of mechanism described above. The profiles of free energy in Figure 1 show that this DMG state corresponds to a broad deep valley on the free-energy landscape of protein, which should work as the funnel directing a folding process. The external boundary of this funnel corresponds to a pseudo-secondary structure of protein with highly loosened interchain hydrogen bonds.

### 3.2. Non-Arrhenius Behavior

#### 3.2.1. Elementary Steps of Protein Unfolding

The calculated rate constants of all three elementary steps of unfolding mechanism (6) demonstrate non-Arrhenius behavior. The Arrhenius plots for these rate constants have a “convex” shape (see Figure 3). This shape corresponds to a strong temperature dependence of the Arrhenius parameters (see Figure 4). As discussed in the Introduction, non-Arrhenius behavior was supposed in literature to be due to the simultaneous contribution of several mechanisms with different Arrhenius parameters. Our results show that non-Arrhenius behavior is characteristic even for elementary steps of the unfolding mechanism (6). Our results show that the main factor of non-Arrhenius behavior is a strong temperature-dependent shift in the location of *DMG* and Transition States on the reaction coordinate (see Figure 5). The shift in Transition State location with temperature was earlier deduced for unimolecular chemical reactions by Quack and Troe [40]. These authors found that the free-energy maximum criterion in canonical Transition State Theory results in the dependence of Transition State location on temperature. The estimated shift in *TS* (free-energy maximum) is illustrated by the results of calculations for dissociation of ethane (see Figure 4 from paper [40]), where the shift in *TS* location is shown for a temperature variation from 300 to 1700 K. The results of our calculations presented in Figure 5 indicate a much stronger temperature effect on the location of free-energy maximum for protein unfolding. This big difference between the cases of *H*-bonds (our case) and covalent bond (case of paper [40]) dissociation is governed by a drastically higher relative contribution of the entropic term to free energy in the case of protein unfolding.

#### 3.2.2. Heat Denaturation in a Wide Temperature Range

The rate of heat denaturation is usually measured as a rate of the loss of folded structure concentration $\frac{d[F]}{dt}$. This rate is governed by the effective rate constant $k_{eff}$, which is equal to the combination of the rate constants of elementary steps. The Arrhenius plot for this rate constant also demonstrates non-Arrhenius behavior (see Figure 6). This plot shows that the Arrhenius parameters for $k_{eff}$ drop drastically with the increase in temperature. Activation energy drops from tens of kcal/mol at a low-temperature limit to units of kcal/mol at high temperature. The preexponential factor drops by nearly 30 orders of magnitude. This drop in the values of the Arrhenius parameters is very similar to the experimental data for several proteins, which are summarized by Sarkar et al. in paper [10]. Sarkar et al. supposed that at high temperature, unfolding is limited by diffusion, which has low activation energy and preexponential factor. Our results indicate that unfolding is limited by the reaction in a wide temperature range. But the values of Arrhenius parameters of reaction change drastically with temperature due to very strong change in the free-energy profile along the reaction coordinate.

The results above were obtained with the use of unfolding enthalpy $∆H_{\infty }$ = 50 kcal/mol as a parameter of the model. Variation in this value does not change the qualitative conclusions, but it has some effect on the quantitative data (see the next subsection).

### 3.3. “Exotic” Values of Arrhenius Parameters

#### 3.3.1. High Preexponential Factor Values

Our results indicate that the maximum values of the preexponential *A*-factor take place in the low-temperature limit of the interval under consideration. The highest values are lg(*A*/s^−1^) ≈ 33 for the rate constant of the individual step of $F\overset{k_{1}}{\to }DMG$ conversion and lg(*A*/s^−1^) ≈ 43 for the rate constant $k_{eff}$ of heat denaturation $F\overset{k_{eff}}{\to }U$. The calculated numbers depend on the value of enthalpy of unfolding $∆H_{\infty }$, which is a parameter of the model. The values of the *A*-factor above are obtained with the value $∆H_{\infty }$ = 50 kcal/mol. Experimental data for protein unfolding demonstrate that many proteins have higher values of unfolding enthalpy up to 600 kJ/mol ≈ 150 kcal/mol and even more [36]. Taking $∆H_{\infty }$ = 150 kcal/mol, we get a rise in the numbers of lg(*A*/s^−1^) up to lg(*A*/s^−1^) ≈ 73 for $F\overset{k_{1}}{\to }DMG$ conversion and lg(*A*/s^−1^) ≈ 103 for heat denaturation $F\overset{k_{eff}}{\to }U$. These high values of *A*-factor for $k_{eff}$ are within the interval of the values observed experimentally for the kinetics of protein denaturation [5,6]. A temperature increase results in a sharp drop in A-values. Figure 4b shows a drop in the *A*-factor in a temperature interval of 270–370 K. At a temperature near 400 K and higher, the channel $F\overset{k_{1}}{\to }DMG$ disappears, because the F state becomes unstable (see Figure 1e,f). The *A*-factor of heat denaturation rate constant $k_{eff}$ also drops at high temperature. The number corresponding to the high-temperature limit of the Arrhenius plot in Figure 6 (lg(*A*/s^−1^) ≈ 11.5) is lower than the typical frequency of molecular vibrations (lg(ν/s^−1^) = 13–14). This drop in *A*-values makes them “non-exotic”. Therefore, the results of our investigation show that the extrapolation of the unfolding rate constant to infinite temperature does not give any “unphysical” results.

#### 3.3.2. Negative Activation Energy

We should also highlight the results indicating the negative activation energy for the rate constant of elementary step $DMG\overset{k_{-1}}{\to }F$ (see Figure 4e). These results can be important for the kinetics of the folding processes, where the step *DMG* → *F* can be the limiting one. Experimental observations of the negative activation energy in the folding processes [41,42,43,44] are in line with the expectations based on our results.

### 3.4. Comments on the Solvent-Free Contribution to the Unfolding Kinetics

Model (6), based on the consideration of unfolding as a breaking of multiple interchain hydrogen bonds, does not contain an interaction of protein with the solvent. Despite this, we can emphasize the high predictive ability of this model, which is confirmed by the successful explanation of many experimental observations. The participation of solvent-free *DMG* structures in the unfolding of proteins in a solution is confirmed by abundant experimental data. The predicted non-Arrhenius behavior and a shape of the Arrhenius plot for a wide temperature range, “exotic” values of the Arrhenius parameters (such as very high preexponential factors for unfolding rate constant, negative activation energy in folding kinetics, et cet.), the enthalpy–entropy compensation and the kinetic isotope effect are in a very good agreement with the experimentally observed phenomena. All these findings allow us to conclude that the solvent-free part of the unfolding reaction coordinate plays a very important role in the kinetics of heat-induced unfolding.

## 4. Methods

Quantum-chemical calculations of the change in structure, electronic energy, vibrational frequencies, zero-point and thermal energies of the formamide dimer in the course of simultaneous dissociation of its two interchain *H*-bonds were carried out with the use of the Gaussian 09 package [45]. The MP2/6–31g(d,p) approach was applied, which was used for calculations of the equilibrium structure of the formamide dimer by Florian and Johnson [20]. Calculations of entropy were carried out according to the Harmonic Oscillator–Rigid Rotor approach. The details and results are presented in Appendix A. The further steps of calculations are shown in the flowchart and detailed below.

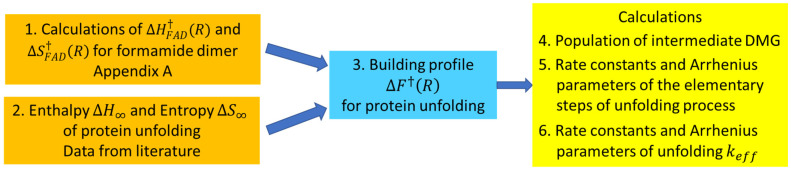

1. The calculations for formamide dimer as a model system give us the functional dependence of enthalpy $∆H_{\mathrm{FAD}}^{†}(R)$ and entropy $∆S_{\mathrm{FAD}}^{†}(R)$ on the *H*-bond distance (*R*). The total enthalpy and entropy of the manifold of breaking *H*-bonds is the sum of these contributions from all breaking *H*-bonds. Therefore, the *R* dependence of total enthalpy and entropy of the manifold of breaking *H*-bonds is functionally the same.
2. The size of the manifold of breaking *H*-bonds is proportional to the value of the enthalpy of unfolding $∆H_{\infty }$, which is used as a parameter in our calculations. The literature data for enthalpy $∆H_{\infty }$ and entropy $∆S_{\infty }$ of unfolding fit the Enthalpy–Entropy Compensation Law (EECL). For our calculations, we took quite typical values of $∆H_{\infty }$ = 50 kcal/mol and an entropy value of $∆S_{\infty }$, corresponding to an experimentally determined linear EECL correlation.
3. The determined R dependence of the enthalpy and entropy of unfolding and the values of $∆H_{\infty }$ and $∆S_{\infty }$ taken from literature allowed us to build the free-energy profile along the reaction coordinate of protein unfolding $∆F^{†}\left(R\right)$
4. The free-energy profile $∆F^{†}\left(R\right)$ built allowed us to reveal the presence of the intermediate assigned to the dry molten globule (DMG) and to calculate its population as a function of temperature, as well as to carry out the TST calculations of the rate constants and the Arrhenius parameters for the elementary steps of unfolding and for unfolding as a whole.

## Data Availability

The data supporting the findings of this study are available within this article.

## Appendix A

### Appendix A.1. Calculations of Enthalpy $∆H_{FAD}^{†}(R)$ and Entropy $∆S_{FAD}^{†}(R)$ for the Formamide Dimer (FAD)

Enthalpy $∆H_{FAD}^{†}(R)$ and entropy $∆S_{FAD}^{†}(R)$ of the formamide dimer (FAD) at a fixed value of reaction coordinate are calculated from their values at the equilibrium distance $R\left(O\cdots H\right)=R_{0}=$1.89 Å, where they are equated to 0. The corresponding equation for enthalpy is:

(A1) $$∆H_{FAD}^{†}(R)=(E\left(R\right)-E\left(R_{0}\right))+{(\langle E^{†}\rangle }_{R}-{\langle E^{†}\rangle }_{R_{0}}),$$

where $E=E_{el}+ZPE$ is the sum of calculated electronic energy and zero-point-energy (ZPE), and ${(\langle E^{†}\rangle }_{R}-{\langle E^{†}\rangle }_{R_{0}})$ is the difference between the values of the average thermal energy at T = 300 K for the dimer at R(O⋯H)=R and $R_{0}$. At long R(O⋯H)= 6 Å, the enthalpy is calculated in a Completely Loose Transition State limit (described in paper [16]):

$$∆H_{FAD}^{†}\left(R=6\;Å\right)=15.3\frac{kcal}{mol}+2\cdot \langle E_{Mon,vib}\rangle +\frac{5}{2}RT-\langle E_{Dim,vib}\rangle =\;13.65\;kcal/mol,$$

where $\langle E_{Mon,vib}\rangle$ and $\langle E_{Dim,vib}\rangle$ are the values of the average vibrational energy of monomer and the dimer of formamide at 300 K, respectively. These numbers are calculated according to the Harmonic Oscillator–Rigid Rotor approximation (HO-RR). The calculated data at the low distance values presented in Figure A1a are limited by the point at *R* = 2.7 Å. At a bigger distance, some harmonic vibrational frequencies become imaginary in values, which does not allow us to apply HO-RR approach. The calculated data for enthalpy are fitted by a Morse potential, which is also presented in Figure A1a.

The equation for entropy is:

(A2) $$∆S_{FAD}^{†}(R)=R\cdot ln\frac{Q^{†}}{Q_{Dim}}+\frac{{(\langle E^{†}\rangle }_{R}-{\langle E^{†}\rangle }_{R_{0}})}{T}$$

The ratio of partition functions is equal to:

$$\frac{Q^{†}}{Q_{dim}}=\frac{Q_{rot}^{†}\cdot Q_{vib}^{†}}{Q_{rot,Dim}\cdot Q_{vib,Dim}},$$

where $Q_{rot,Dim}$ and $Q_{vib,Dim}$ are the rotational and vibrational partition functions of the dimer in equilibrium geometry (*R* = $R_{0}$), $Q_{rot}^{†}$ is the rotational partition function of the dimer at a distance *R*, and $Q_{vib}^{†}$ is the vibrational partition function of the dimer at a fixed distance *R* (the partition function of vibration along the reaction coordinate is excluded). Partition functions are calculated according to the HO-RR approximation. Again, the calculated data at low distance values are limited by the point at *R* = 2.6 Å. For high *R* values, the HO approximation stops working for 5 primarily vibrational degrees of freedom because they convert to the internal rotation. This is taken into account at the high R(O⋯H)=6 Å distance value, where all components of entropy are calculated in a CLTS limit, which gives $∆S^{†}\left(R=6\;Å\right)=\;$22.1 cal/(mol·K). The calculated data for entropy are fitted well by a sigmoidal curve, which is presented in Figure A1b.

### Appendix A.2. Application of the Calculated $∆H_{FAD}^{†}(R)$ and $∆S_{FAD}^{†}(R)$ Interpolations to the Case of Protein Unfolding

According to our model, protein unfolding proceeds as a breaking of a multiple set of interchain *H*-bonds with the reaction coordinate corresponding to a simultaneous change in R(O⋯H) distance for all breaking *H*-bonds. Thus, the total enthalpy $∆H^{†}(R)$ and entropy $∆S^{†}(R)$ of a set of breaking *H*-bonds are higher than but proportional to the determined $∆H_{FAD}^{†}(R)$ and $∆S_{FAD}^{†}(R)$, respectively. The values of interchain R(O⋯H) equilibrium distance in polypeptides or proteins are somewhat higher than the equilibrium value *R ≈* 1.9 Å for the formamide dimer (see Figure A1a). Somewhat longer equilibrium interchain R(O⋯H) distance of 2.1 Å for polyglycine dimer [46] corresponds better to the numbers, characteristic for long chains of proteins. Therefore, for further use, we corrected the interpolation curves for $∆H^{†}(R)$ and $∆S^{†}(R)$ with a slight shift in the *R*_0_ and *R*_1_ values by 0.2 Å, making *R*_0_ = 2.1 Å and *R*_1_ = 2.9 Å. Finally, the interpolation formulae used for unfolding of protein with enthalpy $∆H_{\infty }$ and entropy $∆S_{\infty }$ of denaturation are:

(A3) $$∆H^{†}\left(R\right)=∆H_{\infty }{\left[1-\exp\left(-a\cdot \left(R-R_{0}\right)\right)\right]}^{2},$$

(A4) $$∆S^{†}\left(R\right)=∆S_{\infty }\cdot [\frac{1.17}{1+exp\left(-b\left(R-R_{1}\right)\right)}-0.17],$$

where $∆H_{\infty }$ and $∆S_{\infty }$ are the numbers corresponding to complete unfolding of protein at *R* → ∞. The parameters are *a* = 1.3 Å^−1^, *R*_0_ = 2.1 Å, *b* = 2.2 Å^−1^ and $R_{1}$ = 2.9 Å. In order to choose the numbers of $∆H_{\infty }$ and $∆S_{\infty }$ for model calculations, we referred to the data of the work by Liu et al. [36], who collected the data from the literature on denaturation of 3224 proteins at *T* = 298 K. These data demonstrate a linear correlation, indicating very good enthalpy–entropy compensation. The slope of this linear correlation corresponds to the value of the temperature of complete compensation equal to $T_{c}$ ≈ 330 K. This value allows us to estimate entropy of unfolding at *T* = 300 K:

(A5) $$T\cdot ∆S_{\infty }\approx 0.9\cdot ∆H_{\infty }.$$

A similar ratio between entropy and enthalpy of breaking interchain *H*-bonds was found with quantum-chemical calculations for dissociation of polyglycine dimers of different length carried out in paper [16]. For the current calculations, we took the value $∆H_{\infty }$ = 50 kcal/mol, which corresponds approximately to the middle number of the range of the unfolding enthalpy ($∆H_{un}$) values found experimentally for proteins [36]. Neglecting the weak dependence of $∆S_{\infty }$ and $∆H_{\infty }$ on temperature, and substituting the values of the parameters *a*, *b*, *R*_0_ and *R*_1_, we get the equation for the change in free energy along the reaction coordinate $∆F^{†}\left(R\right)$ at temperature *T*:

$$∆F^{†}\left(R\right)=∆H^{†}\left(R\right)-T\cdot ∆S^{†}\left(R\right)$$

$$\approx ∆H_{\infty }\cdot {\left[1-\exp\left(-a\cdot \left(R-R_{0}\right)\right)\right]}^{2}-T\cdot ∆S_{\infty }\cdot \left[\frac{1.17}{1+exp\left(-b\left(R-R_{1}\right)\right)}-0.17\right]=$$

(A6) $$\approx ∆H_{\infty }\cdot \left\{{\left[1-\exp\left(-1.3\cdot \left(R-2.1\right)\right)\right]}^{2}-0.9\cdot \frac{T}{300}\cdot \left[\frac{1.17}{1+exp\left(-2.2\left(R-2.9\right)\right)}-0.17\right]\right\}.$$

## Appendix B

### Appendix B.1. Constant of Equilibrium F⟺DMG

The ratio of [*DMG*]*_e_* and [*F*]*_e_* concentrations in equilibrium, equal to equilibrium constant *K*_1_, was calculated as a function of temperature via the following equation:

(A7) $$K_{1}=\frac{{\left[DMG\right]}_{e}}{{\left[F\right]}_{e}}=\frac{Q_{DMG}}{Q_{F}}=\frac{{(\int_{R_{2}}^{R_{3}}e^{-\;\frac{F^{†}\left(R\right)}{kT}}dR)}_{DMG}}{{(\int_{R_{1}}^{R_{2}}e^{-\;\frac{F^{†}\left(R\right)}{kT}}dR)}_{F}}$$

where $\frac{Q_{DMG}}{Q_{F}}$ is the ratio of the total partition functions of *DMG* and *F* states. Free-energy $F^{†}\left(R\right)$ profiles at varying temperatures were derived from calculated shape functions f(R) above via Equation (4). The values of $F^{†}\left(R\right)$ in both integrals of Equation (A7) were counted from the common zero. Here, the integration boundary $R_{1}$ corresponds to the distance located on the left slope of f(R) profile for the *F* state in Figure 1b, and boundaries $R_{2}$ and $R_{3}$ correspond to location of Transition States *TS*_1_ and *TS*_2_ in Figure 1b.

### Appendix B.2. Rate Constants of Unfolding Elementary Steps

Rate constants for the steps of mechanism (6) were calculated with the use of TST Equation (2). The locations of the free-energy maxima coincide with maxima on $∆F^{†}\left(R\right)$ profiles for all three processes of scheme (6), corresponding to maxima of the calculated f(R) profiles shown in Figure 1b. But the value $∆F^{\neq }$ in Equation (2) is not equal to the value of $∆F^{†}\left(R\right)$ in this maximum (designated later as$\;∆F^{†}$), because the last term does not contain the contribution of the reactant free energy corresponding to the reaction coordinate itself. Taking this into account, the equation of TST for the rate constant (2) can be rewritten as follows:

(A8) $$k=\frac{k_{B}T}{h}\cdot e^{-\frac{∆F^{\neq }}{RT}}=\frac{k_{B}T}{h}\cdot \frac{1}{Q_{rc,\;r}}e^{-\frac{∆F^{†}}{RT}},$$

where $Q_{rc,\;r}$ is the partition function of the reagent (*F* or *DMG* state dependent on the reaction considered) for the degree of freedom corresponding to the reaction coordinate itself. This partition function is calculated via the following equation:

(A9) $$Q_{rc,r}=\frac{\sqrt{2\pi \mu k_{B}T}}{h}\cdot \int e^{-\;\frac{∆F^{†}(R)}{kT}}dR,$$

where $∆F^{†}(R)$ is counted from zero for the reagent (*F* or *DMG* state). The intervals of *R* for integration correspond to *R*-boundaries for the *F* or *DMG* state. Equation (A9) contains parameter *μ,* corresponding to the reduced mass of two chains of protein which separate in a process of breaking interchain *H*-bonds. We arbitrarily set the value *μ* = 0.5 kDa corresponding to the separation of two chains with mass 1 kDa. This *μ* parameter is the same along the free-energy profile for one system but can differ for different proteins. It is worth noting that this number works as a square root $\sqrt{\mu }$ on the value of the rate constant but does not influence the shapes of any found dependencies of rate constants.
